# Assessment of Cardiovascular Health of Children Ages 6 to 10 Years Conceived by Assisted Reproductive Technology

**DOI:** 10.1001/jamanetworkopen.2021.32602

**Published:** 2021-11-04

**Authors:** Linlin Cui, Min Zhao, Zhirong Zhang, Wei Zhou, Jianan Lv, Jingmei Hu, Jinlong Ma, Mei Fang, Lili Yang, Costan G. Magnussen, Bo Xi, Zi-Jiang Chen

**Affiliations:** 1 Center for Reproductive Medicine, Cheeloo College of Medicine, Shandong University, Jinan, China; 2 Key Laboratory of Reproductive Endocrinology of Ministry of Education, Shandong University, Jinan, China; 3 Shandong Key Laboratory of Reproductive Medicine, Jinan, China; 4 Shandong Provincial Clinical Research Center for Reproductive Health, Jinan, China; 5 National Research Center for Assisted Reproductive Technology and Reproductive Genetics, Shandong University, Jinan, China; 6 Department of Nutrition and Food Hygiene, School of Public Health, Qilu Hospital, Cheeloo College of Medicine, Shandong University, Jinan, China; 7 Children Cardiovascular Research Center, Department of Epidemiology, School of Public Health, Qilu Hospital, Cheeloo College of Medicine, Shandong University, Jinan, China; 8 Menzies Institute for Medical Research, University of Tasmania, Hobart, Australia; 9 Research Centre of Applied and Preventive Cardiovascular Medicine, University of Turku, Turku, Finland; 10 Key Laboratory of Cardiovascular Remodeling and Function Research, Chinese Ministry of Education, Chinese National Health Commission and Chinese Academy of Medical Sciences, State and Shandong Province Joint Key Laboratory of Translational Cardiovascular Medicine, Department of Cardiology, Cheeloo Hospital of Shandong University, Jinan, China; 11 Shanghai Key Laboratory for Assisted Reproduction and Reproductive Genetics, Shanghai, China; 12 Center for Reproductive Medicine, Renji Hospital, School of Medicine, Shanghai Jiao Tong University, Shanghai, China

## Abstract

**Importance:**

Assisted reproductive technology (ART) has been widely used for treatment of infertility and has brought millions of births worldwide. The health of offspring conceived by ART has been of much concern, and adverse cardiovascular health outcomes have been reported by previous studies.

**Objective:**

To assess the cardiovascular health of children conceived by ART.

**Design, Setting, and Participants:**

This cohort study was conducted among participants recruited from November 2017 to February 2019. Participants were 382 children conceived by ART who were selected from a single reproductive center and 382 children who were naturally conceived, randomly selected from a primary school, and matched by sex, age, and maternal age at the child’s birth (2 years older or younger). Data were analyzed from March 2019 through December 2019.

**Exposures:**

Conception by ART.

**Main Outcomes and Measures:**

Blood pressure was measured, and echocardiography was performed to determine left ventricular structural and functional parameters. Adjusted relative wall thickness (aRWT) was found for age, with high RWT defined as an aRWT of 0.375 or more.

**Results:**

Among 764 children aged 6 to 10 years, 382 children were conceived by ART (mean [SD] age, 7.20 [1.21] years; 201 [52.6%] boys) and 382 children were naturally conceived (mean [SD] age, 7.20 [1.21] years; 201 [52.6%] boys). Children conceived by ART had statistically significantly increased mean (SD) height (130.2 [9.5] cm vs 128.5 [8.1] cm; *P* = .007) and body mass index (17.6 [3.6] vs 17.1 [2.7]; *P* = .03). Those conceived by ART, compared with children in the matched control group, had statistically significantly increased blood pressure (mean [SD] systolic blood pressure, 105.5 [6.9] mm Hg vs 103.5 [8.4] mm Hg; adjusted *P* < .001; mean [SD] diastolic blood pressure, 67.2 [5.6] mm Hg vs 62.2 [6.3] mm Hg ; adjusted *P* < .001), left ventricular systolic dysfunction (mean [SD] left ventricular ejection fraction, 64.61% [3.20%] vs 66.70% [3.89%]; adjusted *P* < .001), and diastolic dysfunction (mean [SD] early/late mitral/tricuspid diastolic velocities ratio, 1.66 [0.28] vs 2.21 [0.36]; adjusted *P* < .001). They also had statistically significantly increased parameters of left ventricular structure, including mean (SD) left ventricular mass index (31.97 [5.04] g/m^2.7^ vs 28.28 [3.54] g/m^2.7^; adjusted *P* < .001) and RWT (3.30 [0.41] mm vs 2.98 [0.14] mm; adjusted *P* < .001). Additionally, children conceived by ART had statistically significantly increased prevalence of left ventricular hypertrophy (9 children [2.4%] vs 2 children [0.5%]; *P* = .03), high RWT (61 children [16.0%] vs 0 children; *P* < .001), and left ventricle remodeling patterns, including concentric remodeling (60 children [15.7%] vs 0 children), eccentric hypertrophy (8 children [2.1%] vs 2 children [0.5%]), and concentric hypertrophy (1 child [0.3%] vs 0 children) (*P* for left ventricle remodeling < .001).

**Conclusions and Relevance:**

This study found that children conceived by ART had increased blood pressure and unfavorable changes in left ventricular structure and function compared with children who were naturally conceived. These findings suggest that further studies are needed to investigate the potential mechanisms and long-term outcomes associated with these differences.

## Key Points

**Question:**

Is conception assisted by reproductive technology associated with offspring cardiovascular health?

**Findings:**

In this cohort study of 764 children born with or without assisted reproductive technology, children conceived by assisted reproductive technology had statistically significantly worse outcomes in left ventricular function and structure.

**Meaning:**

These findings suggest the importance of early detection, potential interventions, and improvement of cardiovascular health among offspring conceived by assisted reproductive technology.

## Teaser

This cohort study examines the association of conception by assisted reproductive technology with cardiovascular health outcomes among children in China ages 6-10 years.

## Introduction

Assisted reproductive technology (ART) is widely used in the treatment of infertility. It is estimated that more than 8 million infants have been born after the use of ART worldwide since Louise Brown’s birth, accounting for approximately 2% to 6% of births in high-income countries.^1,2^ ART requires the in vitro manipulation of gametes and embryos in a synthetic culture environment, and these nonphysiological exposures may be associated with adverse outcomes in embryonic development and offspring health.^3^ Some studies^4,5^ found that ART may be associated with increased risk of unfavorable obstetric outcomes, including preterm birth, low birth weight, and infants born small for their gestational age. These are also risk factors associated with cardiovascular disease.^6,7^ Evidence from the developmental origins of health and disease theory also suggests that adverse events (eg, restricted fetal growth and preterm birth) in early life are associated with increased the risk of cardiovascular disease later in life.^8^ Therefore, the cardiovascular health of offspring conceived by ART has gained much attention. Some scholars have found an increased incidence of congenital heart defects among children conceived by ART,^9^ and cardiac remodeling already presents in fetal life^10^ and persists in postnatal life and at ages 2 to 6 years among offspring conceived by ART.^10,11,12^ However, other studies did not find an increased risk of cardiovascular disease among children conceived by ART.^13,14^ These studies include limited sample sizes, and the results were inconsistent.^11,12,15^ Additionally, measurement of the heart among children aged 2 to 6 years may be inaccurate because of their low compliance to examination and relatively small heart size. Therefore, to investigate the association of ART with heart health outcomes, we used cardiology ultrasonography measurements to assess left ventricular cardiac structure and function among children aged 6 to 10 years conceived by ART compared with children in a matched control group conceived naturally.

## Methods

This cohort study’s protocol was approved by the institutional review board of the Center for Reproductive Medicine of Shandong University. Written informed consent was obtained from parents or guardians of all participants. This report followed the Strengthening the Reporting of Observational Studies in Epidemiology (STROBE) reporting guideline for cohort studies.

This study including 382 children conceived by ART (ie, born after intracytoplasmic sperm injection [ICSI] or in vitro fertilization [IVF]) and 382 children in a matched control group aged 6 to 10 years. From November 2017 to February 2019, based on register records from pregnancies conceived by ART at the Center for Reproductive Medicine in Shandong Provincial Hospital, affiliated to Shandong University, 435 children who were conceived by ART and their parents or guardians were invited through the phone initially, with 382 parents or guardians agreeing to participate in the study. Children in the naturally conceived control group were randomly selected from 1 primary school in Shandong, China. Children conceived by ART were matched 1 to 1 with children in the naturally conceived control group by sex, age, and maternal age at the child’s birth (ie, within 2 years).

### Physical and Lifestyle Measurements

Standardized protocols and instruments were used for all measurements among children conceived by ART and those in the matched control group. Weight and height were measured with children in light clothes and without shoes using a standardized scale. Weight was measured twice to the nearest 0.1 kg, and height was measured twice to the nearest 0.1 cm. The means of the 2 measures were calculated for data analysis. Body mass index (BMI) was calculated as weight in kilograms divided by height in meters squared. Overweight and obesity were defined using age-specific and sex-specific BMI percentile cutoffs for Chinese children and adolescents.^16^

Blood pressure (BP) was measured by trained staff (Z.Z., J.H., J.M., and M.F.) with an electronic device (Omron HEM-7012; Omron), which has been clinically validated.^17^ After the child had at least 10 minutes of rest, BP was measured on the right arm using the appropriate-sized cuff, with the child seated. We obtained 3 consecutive BP measurements, and the mean value of the final 2 readings was used for data analysis. Elevated BP and high BP were defined as systolic BP (SBP) or diastolic BP (DBP) at or above the age-specific and sex-specific 90th and 95th percentile values, respectively, of the Chinese pediatric BP references.^18^ Lifestyle factors, including frequency of fruit and vegetable intake, soft drink intake, physical activity (ie, low to moderate or vigorous exercise), and duration of screen time (ie, watching TV or playing computer games) and night sleep, were collected using the same questionnaire.

### Cardiology Ultrasonography Measurements and Definitions

For all participants, color Doppler ultrasonography examination of the LV was performed using a portable ultrasonography machine with an S4-2 linear transducer of 2 to 4 MHz (CX30, Philips). Left ventricular end-diastolic internal dimension (LVDD), end-systolic internal dimension (LVSD), interventricular septal thickness (IVST), and left ventricular posterior wall thicknesses (LVPWT) were measured. Left ventricular mass (LVM) was calculated using the Devereux formula^19^: LVM (g) = 0.8 × [1.04 × (LVDD + IVST + LVPWT)^3^ − (LVDD)^3^] + 0.6. Left ventricular mass index (LVMI) was calculated as LVM (in grams) divided by height (in meters)^2.7^ to correct for body size.^20^ Left ventricular hypertrophy (LVH) was defined as LVMI at or above the age-specific and sex-specific 95th percentile based on 2273 US children who were healthy and did not have obesity.^21^ Relative wall thickness (RWT) was calculated as (LVPWT + IVST) / LVDD. We then found adjusted RWT (aRWT) for age using the formula aRWT = RWT − 0.005 × (age − 10) for children aged 1 to 17 years.^22^ High RWT was defined as an aRWT of 0.375 or more, which was the age-specific 95th percentile cutoff for aRWT.^22^ Based on LVMI and aRWT, children were then divided into 4 groups: reference range geometry (ie, reference range LVMI and aRWT), concentric remodeling (CR; reference range LVMI and high aRWT), eccentric hypertrophy (EH; LVH and reference range aRWT), and concentric hypertrophy (CH; LVH and high aRWT).

The LV shortening fraction (LVSF) was calculated using internal ventricular diameters with the following equation: (LVDD − LVSD) / LVDD. Mitral inflow velocities, including peak velocities during early diastole (E) and late diastole (A), were measured, and E to A ratio was calculated.

### Statistical Analysis

All data analyses were performed using SAS statistical software version 9.3 (SAS Institute). Study outcomes included BP and cardiac structure and function. The independent variable of interest was conception type (ART vs natural), and the covariates were gestational age at birth, birth weight, duration of breastfeeding, current (ie, at age 6-10 years) weight status, BP status, and lifestyle factors, including fruit and vegetable intake, soft drink intake, physical activity, and duration of screen time and sleep. Data were expressed as mean (SD) for continuous variables (all variables were in approximately normal distribution) and No. (%) for categorical variables. Early life factors, current weight, and lifestyle factors were compared between ART and control populations using *t* test or χ^2^ test, as appropriate. The parameters of BP indices, cardiac structure, and function were compared between groups using *t* test. To rule out the association of covariates with outcomes, linear regression analysis was used to assess the associations of conception type (ie, ART vs natural) with BP indices and cardiac structure and function after adjustment for gestational age at birth, birth weight, duration of breastfeeding, current weight status, BP status (for outcomes of cardiac structure and function), and lifestyle factors. Additionally, given that it is hypothesized that fertilization type (ie, ICSI vs IVF) and embryo type (ie, fresh vs frozen) may have different associations with LV structure and function,^23,24^ we performed subgroup analyses by fertilization type and embryo type. The prevalence of LVH, elevated BP, high RWT, and LV remodeling patterns were compared between groups using χ^2^ test or Fisher exact test. A 2-sided *P* < .05 was considered statistically significant. Data were analyzed from March 2019 through December 2019.

## Results

### Characteristics of Study Groups

Among 764 children aged 6 to 10 years enrolled in the study, 382 children were conceived by ART (mean [SD] age, 7.20 [1.21] years; 201 [52.6%] boys) and 382 children were naturally conceived (mean [SD] age, 7.20 [1.21] years; 201 [52.6%] boys) (Table 1). Mean (SD) maternal age at the child’s birth was 30.49 (4.34) years for children conceived by ART and 31.11 (3.92) years for children in the control group. All participants were singletons and free of genetic, cardiovascular, and endocrine diseases. Children conceived by ART had statistically significantly increased current mean (SD) height (130.2 [9.5] cm vs 128.5 [8.1] cm; *P* = .007), weight (30.5 [10.2] kg vs 28.5 [7.1] kg; *P* = .003), and BMI (17.6 [3.6] vs 17.1 [2.7]; *P* = .03) but similar prevalence of overweight and obesity compared with children who were naturally conceived. Statistically significant differences between groups were also found in early life factors, including increased birth weight and longer breastfeeding duration among children conceived by ART. A statistically significantly greater proportion of children conceived by ART consumed soft drinks 1 or more times per week and had 2 or more hours of screen time per day. However, the distribution of gestational age at birth (ie, proportion of children born preterm, at full term, or postterm), current frequency of fruit and vegetable intake, physical activity, and mean sleep duration were similar between groups.

**Table 1.** Participant Characteristics by Conception Type

Characteristic^a^	Children (N = 764)		*P* value
	Control group (n = 382)	ART group (n = 382)	
Early life factors			
Age, mean (SD), y	7.2 (1.2)	7.2 (1.2)	NA
Gestational age at birth, No. (%)			
<37 wk (preterm)	20 (5.2)	20 (5.2)	.15
37-42 wk (full-term)	352 (92.2)	359 (94.0)	
>42 wk (postterm)	10 (2.6)	3 (0.8)	
Birth weight, mean (SD), g	3384 (432)	3464 (491)	.017
Birth weight category, No. (%)			
<2500 g	7 (1.8)	11 (2.9)	.03
2500-4000 g	341 (89.3)	316 (82.7)	
≥4000 g	34 (8.9)	55 (14.4)	
Duration of breastfeeding, No. (%)			
Never	6 (1.6)	14 (3.7)	<.001
0-3 mo	6 (1.6)	21 (5.5)	
3-5 mo	25 (6.5)	17 (4.5)	
6-12 mo	147 (38.5)	56 (14.7)	
>12 mo	198 (51.8)	274 (71.7)	
Current body size measures			
Height, mean (SD), cm	128.5 (8.1)	130.2 (9.5)	.007
Weight, mean (SD), kg	28.5 (7.1)	30.5 (10.2)	.003
BMI, mean (SD)	17.1 (2.7)	17.6 (3.6)	.03
Weight status, No. (%)			
Overweight	62 (16.2)	63 (16.5)	.44
Obesity	78 (20.4)	92 (24.1)	
Current lifestyle factors			
≥5 times/d of fruit and vegetable intake, No. (%)	64 (16.8)	58 (15.2)	.55
≥1 times/wk soft drink intake, No. (%)	21 (5.5)	41 (10.7)	.008
≥1 h/d physical activity, No. (%)	143 (38.3)	147 (38.5)	.97
≥2 h/d screen time, No. (%)	42 (11.1)	88 (23.0)	<.001
Sleep duration, mean (SD), h/d	9.3 (0.5)	9.4 (0.6)	.38

Abbreviations: ART, assisted reproductive technology; BMI, body mass index (calculated as weight in kilograms divided by height in meters squared); NA, not applicable.

^a^ Categorical variables are presented as No. (%), and continuous variable are presented as mean (SD).

### Blood Pressure and LV Structure and Function

Children conceived by ART, compared with children who were naturally conceived, had statistically significantly increased mean (SD) SBP (105.5 [6.9] mm Hg vs 103.5 [8.4] mm Hg; mean difference, 2.0 mm Hg [95% CI, 0.9-3.0 mm Hg]; adjusted *P* < .001) and DBP (67.2 [5.6] mm Hg vs 62.2 [6.3] mm Hg; mean difference, 5.0 mm Hg [95% CI, 4.1-5.8 mm HG]; adjusted *P* < .001). The prevalence of elevated BP (80 children [20.9%] vs 50 children [13.1%]) and high BP (69 children [18.1%] vs 54 children [14.1%]) was also increased in the ART group vs the control group (*P* for BP status = .002) (Table 2).

**Table 2.** Cardiovascular Outcomes at Ages 6 to 10 Years by Conception Type

Outcome	Mean (SD)		*P* value	Mean difference (95% CI)
	Control group (n = 382)	ART group (n = 382)		
BP level^a^				
SBP, mm Hg	103.5 (8.4)	105.5 (6.9)	<.001	2.0 (0.9 to 3.0)
DBP, mm Hg	62.2 (6.3)	67.2 (5.6)	<.001	5.0 (4.1 to 5.8)
LV structure^a^				
LVDD, mm	38.12 (1.93)	38.61 (3.87)	.05	0.49 (0.05 to 0.92)
LVSD, mm	24.26 (1.66)	25.21 (2.79)	<.001	0.95 (0.62 to 1.28)
LVPWT, mm	5.75 (0.44)	6.28 (0.47)	<.001	0.53 (0.46 to 0.59)
IVST, mm	5.60 (0.43)	6.34 (0.43)	<.001	0.74 (0.68 to 0.81)
LVM, g	55.75 (10.59)	65.30 (14.54)	<.001	9.55 (7.74 to 11.36)
LVMI, g/m^2.7^	28.28 (3.54)	31.97 (5.04)	<.001	3.69 (3.07 to 4.31)
RWT, mm	2.98 (0.14)	3.30 (0.41)	<.001	0.32 (0.28 to 0.37)
LV function^a^				
LVEF, %	66.70 (3.89)	64.61 (3.20)	<.001	−2.09 (−2.60 to −1.59)
LVSF, %	36.37 (3.01)	34.74 (2.82)	<.001	−1.63 (−2.04 to −1.21)
E/A ratio	2.21 (0.36)	1.66 (0.28)	<.001	−0.55 (−0.60 to −0.50)
BP status, No. (%)^b^				
Elevated BP	50 (13.1)	80 (20.9)	.002	NA
High BP	54 (14.1)	69 (18.1)		
LVH, No. (%)^b^	2 (0.5)	9 (2.4)	.03	NA
High RWT, No. (%)^b^	0	61 (16.0)	<.001	NA
LV remodeling patterns, No. (%)^b^				
Concentric remodeling	0	60 (15.7)	<.001	NA
Eccentric hypertrophy	2 (0.5)	8 (2.1)		
Concentric hypertrophy	0	1 (0.3)		

Abbreviations: ART, assisted reproductive technology; BP, blood pressure; DBP, diastolic blood pressure; E/A, early to late mitral/tricuspid diastolic velocities; IVST, interventricular septal thickness; LV, left ventricle; LVDD, left ventricular end-diastolic internal dimension; LVEF, left ventricular ejection fraction; LVH, left ventricular hypertrophy; LVM, left ventricular mass; LVMI, left ventricular mass index; LVPWT, left ventricular posterior wall thickness at end diastole; LVSD, left ventricular end systolic internal dimension; LVSF, left ventricular shortening fraction; RWT, relative wall thickness; SBP, systolic blood pressure.

^a^ *P* values were based on linear regression models adjusted for gestational age at birth, birth weight, duration of breastfeeding, current body mass index, blood pressure status, and current lifestyle factors (ie, fruit and vegetable intake, soft drink intake, physical activity, screen time, and sleep duration).

^b^ *P* values were based on χ^2^ test or Fisher exact test.

Children conceived by ART, compared with children who were naturally conceived, had increased mean (SD) LVSD (25.21 [2.79] mm vs 24.26 [1.66] mm; mean difference, 0.95 mm [95% CI, 0.62 to 1.28 mm]; adjusted *P* < .001), LVPWT (6.28 [0.47] mm vs 5.75 [0.44] mm; mean difference, 0.53 mm [95% CI, 0.46 to 0.59 mm]; adjusted *P* < .001), IVST (6.34 [0.43] mm vs 5.60 [0.43] mm; mean difference, 0.74 mm [95% CI, 0.68 to 0.81 mm]; adjusted *P* < .001), LVM (65.30 [14.54] g vs 55.75 [10.59] g; mean difference, 9.55 g [95% CI, 7.74 to 11.36 g]; adjusted *P* < .001), LVMI (31.97 [5.04] g/m^2.7^ vs 28.28 [3.54] g/m^2.7^; mean difference 3.69 g/m^2.7^ [95% CI, 3.07 to −4.31 g/m^2.7^]; adjusted *P* < .001); and RWT, (3.30 [0.41] mm vs 2.98 [0.14] mm; mean difference, 0.32 mm [95% CI, 0.28 to 0.37 mm]; *P* < .001), which remained significant after adjustment for early life factors, current weight status, BP status, and current lifestyle factors (Table 2). Children conceived by ART had statistically significantly decreased mean (SD) LVEF (64.61% [3.20%] vs 66.70% [3.89%]; mean difference, −2.09 percentage points [95% CI, −2.60 to −1.59 percentage points]; adjusted *P* < .001), LVSF (34.74% [2.82%] vs 36.37% [3.01%]; mean difference, −1.63 percentage points [95% CI, −2.04 to −1.21 percentage points]; adjusted *P* < .001), and E to A ratio (1.66 [0.28] vs 2.21 [0.36]; mean difference, −0.55 [95% CI, −0.60 to −0.50]; adjusted *P* < .001) compared with children in the control group (Table 2). There were similar associations with cardiac indices among children conceived by ART, compared with matched controls, when stratified by fertilization and embryo types (eTable 1 and eTable 2 in the Supplement).

Among children conceived by ART, compared with children in the control group, there was a statistically significantly increased prevalence of LVH (9 children [2.4%] vs 2 children [0.5%]; *P* = .03), high RWT (61 children [16.0%] vs 0 children; *P* < .001), and LV geometric remodeling patterns (CR: 60 children [15.7%] vs 0 children; EH: 8 children [2.1%] vs 2 children [0.5%]; CH: 1 child [0.3%] vs 0 children; *P* < .001) (Table 2). Similar results were found when children conceived by ART were stratified by fertilization type (ie, ICSI or IVF) (eFigure 1 in the Supplement). However, frozen embryo transfer, compared with the control group, had a similar prevalence of LVH, EH, and CH but a statistically significantly increased prevalence of high RWT and CR. In contrast, fresh embryo transfer had statistically significantly increased prevalence of LVH, high RWT, and LV geometric remodeling compared with the control group (eFigure 2 in the Supplement).

## Discussion

To our knowledge, this cohort study is the largest study (with 382 children conceived by ART and 382 children matched in the control group) to examine the associations of ART with alterations in LV structure and function among Chinese children aged 6 to 10 years. Our study found that abnormal cardiac structure and dysfunction, including an increased risk of LVH and LV geometric remodeling, were more frequent among children conceived by ART compared with children in the control group. These findings were independent of early life factors, current lifestyle factors, and fertilization type (ie, ICSI or IVF). However, frozen embryo transfer had some favorable cardiac outcomes compared with fresh-embryo transfer, which may be associated with decreased risks of several unfavorable birth outcomes (eg, being born small for gestational age, low birth weight, and preterm delivery) that are associated with pregnancies conceived from frozen embryo transfer vs fresh embryo transfer.^25^

Several studies have consistently found that children conceived by ART had increased carotid intima-media thickness, increased pulse wave velocity, and decreased flow-mediated dilation.^10,26,27,28^ However, 3 studies^11,12,15^ examining the associations of ART with LV structure and function in childhood found inconsistent results. In a 2014 study among 128 children conceived by ART (ages 2-6 years) and 100 children in a control, Zhou et al^11^ reported that LV geometric morphology remodeling and diastolic dysfunction, but not systolic dysfunction, were increased among children conceived by ART compared with children in the control group. In 2015, Liu et al^12^ found that systolic and diastolic dysfunction were present but alterations in LV morphometry were absent among children conceived by ART. However, a study^15^ among 54 children conceived by ART and 54 children in a control group found significant differences in cardiac structure and function only under stressful conditions of high-altitude–induced pressure overload and hypoxia. Our study, using a larger sample size than previous studies, found that clinically important alterations in LV structure and function were present at significantly increased levels among children aged 6 to 10 years conceived by ART.

Evidence from the Framingham Offspring Study^29^ suggests that LVM can persist from childhood to adulthood and may be a factor associated with cardiovascular disease in adulthood. There are 3 forms of LV geometric remodeling patterns (CR, EH, and CH) that are associated with risk of cardiovascular disease, while CH is considered to carry the greatest risk for cardiovascular disease.^30,31^ To our knowledge, our study is the first to assess the associations of ART with LVH and LV geometric remodeling. We found that children conceived by ART had increased prevalence of LVH, high RWT, and LV geometric remodeling patterns (ie, CR, EH, and CH). We also found that CR was the most frequent pattern of LV geometric remodeling among children conceived by ART.

There are several potential mechanisms that may explain the association of ART with abnormal LV structure and dysfunction. First, epigenetic perturbations induced by ART during the periconceptional period may play an important role in the fetal programming of future subclinical cardiovascular alterations.^32,33,34^ Several studies found that ART may be associated with altered methylation levels among differential methylation regions of imprinted genes in the embryo and placenta.^35,36,37,38^ The incidence of imprinting disorders among offspring conceived by ART is increased compared with offspring conceived spontaneously.^39,40^ These epigenetic alterations may be associated with the infertility backgrounds of parents, superovulation, or factors associated with the ART procedure.^41,42^ The alterations may persist into childhood and be associated with worsened cardiovascular health among offspring.^33,34^ Second, increased oxidative stress (OS) may also be associated with altered cardiac structure and function among offspring conceived by ART. Several publications suggested that OS was crucial in the fetal programming of adulthood cardiovascular diseases.^43,44,45,46^ The infertility backgrounds of parents, pregnancy complications, and the ART procedure could be associated with excessive OS, which may be associated with the cardiac health of offspring.^47^ Third, it has been shown that ART may be associated with increased risk of impaired lipid and glucose metabolism, which is the pathophysiological basis of various cardiovascular diseases.^48,49,50,51,52,53,54,55^ Therefore, ART may be associated with adverse LV structure and function among offspring through the disorder of lipid and glucose metabolism.

### Limitations

Although we conducted a strict matched cohort study with a large sample size and adjusted for many confounders, several limitations of this study should still be noted. First, we cannot make conclusions concerning whether the observed association was associated with the ART procedure itself or other unknown confounders that were not measured in the present study. However, a previous study’s findings^33^ suggested that the ART procedure itself, rather than parent-associated factors, may be the main factor associated with this outcome. Second, it should be noted that because there are no unified criteria to define LVH, high RWT, or LV geometric remodeling patterns, we used sex-specific and age-specific 95th percentile cutoffs based on definitions established among Western children,^21,22^ and the cutoffs may be too high to be used among Chinese children. Third, all children conceived by ART in this study were from a single medical center in Shandong, China; therefore, our findings should be generalized with caution. Additionally, although the differences between groups were significant, the absolute values of the changed parameters in the ART group were still within the reference range, which may limit the clinical relevance of our findings. However, considering the association between the cardiovascular parameter changes in childhood and adult cardiovascular disorders, a continuous monitoring was suggested in this ART-conceived population.

## Conclusions

This study found increased levels of unfavorable changes in LV structure and function, including LV geometric remodeling, among children conceived by ART compared with children who were naturally conceived. These findings may have significance for clinical and public health. Because childhood is a critical window for early detection, potential intervention, and improvement of cardiac health among children conceived by ART, it may be possible to reverse the unfavorable alterations in their cardiac structure and function. Further studies are necessary to confirm the association between ART and cardiovascular remodeling and to explore the potential biological mechanisms underlying the association.

## Notes

Supplement. **eTable 1.** Left Ventricular Structure and Function at Ages 6-10 Years by Assisted Reproductive Technology Fertilization Groups and Control Group **eTable 2.** Left Ventricular Structure and Function at Ages 6-10 Years by Embryo Groups and Control Group **eFigure 1.** Prevalence of Left Ventricular Hypertrophy (LVH), High Relative Wall Thickness (RWT), and Left Ventricular Remodeling by Fertilization Groups and Control Group **eFigure 2.** Prevalence of Left Ventricular Hypertrophy (LVH), High Relative Wall Thickness (RWT), and Left Ventricular Remodeling by Embryo Group and Control Group

## References

[1] Crawford GE, Ledger WL. In vitro fertilisation/intracytoplasmic sperm injection beyond 2020. BJOG. 2019;126(2):237-243. doi:10.1111/1471-0528.15526 30548407

[2] Calhaz-Jorge C, De Geyter C, Kupka MS, ; European IVF-monitoring Consortium (EIM); European Society of Human Reproduction and Embryology (ESHRE). Assisted reproductive technology in Europe, 2013: results generated from European registers by ESHRE. Hum Reprod. 2017;32(10):1957-1973. doi:10.1093/humrep/dex264 29117383

[3] Vrooman LA, Bartolomei MS. Can assisted reproductive technologies cause adult-onset disease: evidence from human and mouse. Reprod Toxicol. 2017;68:72-84. doi:10.1016/j.reprotox.2016.07.015 27474254 PMC5269604

[4] Qin J, Liu X, Sheng X, Wang H, Gao S. Assisted reproductive technology and the risk of pregnancy-related complications and adverse pregnancy outcomes in singleton pregnancies: a meta-analysis of cohort studies. Fertil Steril. 2016;105(1):73-85.e1, 6. doi:10.1016/j.fertnstert.2015.09.007 26453266

[5] Chen M, Heilbronn LK. The health outcomes of human offspring conceived by assisted reproductive technologies (ART). J Dev Orig Health Dis. 2017;8(4):388-402. doi:10.1017/S2040174417000228 28416029

[6] Kaijser M, Bonamy AK, Akre O, . Perinatal risk factors for ischemic heart disease: disentangling the roles of birth weight and preterm birth. Circulation. 2008;117(3):405-410. doi:10.1161/CIRCULATIONAHA.107.710715 18172034

[7] de Jong F, Monuteaux MC, van Elburg RM, Gillman MW, Belfort MB. Systematic review and meta-analysis of preterm birth and later systolic blood pressure. Hypertension. 2012;59(2):226-234. doi:10.1161/HYPERTENSIONAHA.111.181784 22158643 PMC3266458

[8] Hanson M, Gluckman P. Developmental origins of noncommunicable disease: population and public health implications. Am J Clin Nutr. 2011;94(6)(suppl):1754S-1758S. doi:10.3945/ajcn.110.001206 21525196

[9] Patil AS, Nguyen C, Groff K, Wu J, Elliott J, Gunatilake RP. Severity of congenital heart defects associated with assisted reproductive technologies: case series and review of the literature. Birth Defects Res. 2018;110(8):654-661. doi:10.1002/bdr2.1228 29714054

[10] Valenzuela-Alcaraz B, Crispi F, Bijnens B, . Assisted reproductive technologies are associated with cardiovascular remodeling in utero that persists postnatally. Circulation. 2013;128(13):1442-1450. doi:10.1161/CIRCULATIONAHA.113.002428 23985787

[11] Zhou J, Liu H, Gu HT, . Association of cardiac development with assisted reproductive technology in childhood: a prospective single-blind pilot study. Cell Physiol Biochem. 2014;34(3):988-1000. doi:10.1159/000366315 25201231

[12] Liu H, Zhang Y, Gu HT, . Association between assisted reproductive technology and cardiac alteration at age 5 years. JAMA Pediatr. 2015;169(6):603-605. doi:10.1001/jamapediatrics.2015.0214 25915111

[13] Halliday J, Lewis S, Kennedy J, . Health of adults aged 22 to 35 years conceived by assisted reproductive technology. Fertil Steril. 2019;112(1):130-139. doi:10.1016/j.fertnstert.2019.03.001 31003618

[14] Shiloh SR, Sheiner E, Wainstock T, . Long-term cardiovascular morbidity in children born following fertility treatment. J Pediatr. 2019;204:84-88.e2. doi:10.1016/j.jpeds.2018.08.070 30291022

[15] von Arx R, Allemann Y, Sartori C, . Right ventricular dysfunction in children and adolescents conceived by assisted reproductive technologies. J Appl Physiol (1985). 2015;118(10):1200-1206. doi:10.1152/japplphysiol.00533.2014 25979934

[16] National Health Commission of the People's Republic of China. WS/T 586–2018 Screening for Overweight and Obesity Among School-Age Children and Adolescents. Standards Press of China; 2018.

[17] Meng LH, Hou DQ, Shan XY, Mi J. Accuracy evaluation of Omron HEM-7012 electronic sphygmomanometers in measuring blood pressure of children and adolescents. Article in Chinese. Chin J Hypertens. 2013;21:158-162. doi:10.16439/j.cnki.1673-7245.2013.02.036

[18] Fan H, Yan Y, Mi J. Updating blood pressure references for Chinese children aged 3-17 years. Article in Chinese. Chin J Hypertens. 2017;25:428-435. doi:10.16439/j.cnki.1673-7245.2017.05.010

[19] Devereux RB, Alonso DR, Lutas EM, . Echocardiographic assessment of left ventricular hypertrophy: comparison to necropsy findings. Am J Cardiol. 1986;57(6):450-458. doi:10.1016/0002-9149(86)90771-X 2936235

[20] de Simone G, Daniels SR, Devereux RB, . Left ventricular mass and body size in normotensive children and adults: assessment of allometric relations and impact of overweight. J Am Coll Cardiol. 1992;20(5):1251-1260. doi:10.1016/0735-1097(92)90385-Z 1401629

[21] Khoury PR, Mitsnefes M, Daniels SR, Kimball TR. Age-specific reference intervals for indexed left ventricular mass in children. J Am Soc Echocardiogr. 2009;22(6):709-714. doi:10.1016/j.echo.2009.03.003 19423289

[22] de Simone G, Daniels SR, Kimball TR, . Evaluation of concentric left ventricular geometry in humans: evidence for age-related systematic underestimation. Hypertension. 2005;45(1):64-68. doi:10.1161/01.HYP.0000150108.37527.57 15557389

[23] Berntsen S, Söderström-Anttila V, Wennerholm UB, . The health of children conceived by ART: ‘the chicken or the egg?’. Hum Reprod Update. 2019;25(2):137-158. doi:10.1093/humupd/dmz001 30753453

[24] Shi Y, Sun Y, Hao C, . Transfer of fresh versus frozen embryos in ovulatory women. N Engl J Med. 2018;378(2):126-136. doi:10.1056/NEJMoa1705334 29320646

[25] Maheshwari A, Pandey S, Amalraj Raja E, Shetty A, Hamilton M, Bhattacharya S. Is frozen embryo transfer better for mothers and babies: can cumulative meta-analysis provide a definitive answer? Hum Reprod Update. 2018;24(1):35-58. doi:10.1093/humupd/dmx031 29155965

[26] Scherrer U, Rimoldi SF, Rexhaj E, . Systemic and pulmonary vascular dysfunction in children conceived by assisted reproductive technologies. Circulation. 2012;125(15):1890-1896. doi:10.1161/CIRCULATIONAHA.111.071183 22434595

[27] Meister TA, Rimoldi SF, Soria R, . Association of assisted reproductive technologies with arterial hypertension during adolescence. J Am Coll Cardiol. 2018;72(11):1267-1274. doi:10.1016/j.jacc.2018.06.060 30190005

[28] Valenzuela-Alcaraz B, Serafini A, Sepulveda-Martínez A, . Postnatal persistence of fetal cardiovascular remodelling associated with assisted reproductive technologies: a cohort study. BJOG. 2019;126(2):291-298. doi:10.1111/1471-0528.15246 29673050

[29] Lieb W, Xanthakis V, Sullivan LM, . Longitudinal tracking of left ventricular mass over the adult life course: clinical correlates of short- and long-term change in the Framingham Offspring Study. Circulation. 2009;119(24):3085-3092. doi:10.1161/CIRCULATIONAHA.108.824243 19506113 PMC2761217

[30] Verdecchia P, Angeli F, Achilli P, . Echocardiographic left ventricular hypertrophy in hypertension: marker for future events or mediator of events? Curr Opin Cardiol. 2007;22(4):329-334. doi:10.1097/HCO.0b013e3280ebb413 17556886

[31] Muiesan ML, Salvetti M, Monteduro C, . Left ventricular concentric geometry during treatment adversely affects cardiovascular prognosis in hypertensive patients. Hypertension. 2004;43(4):731-738. doi:10.1161/01.HYP.0000121223.44837.de 15007041

[32] Lane M, Robker RL, Robertson SA. Parenting from before conception. Science. 2014;345(6198):756-760. doi:10.1126/science.1254400 25124428

[33] Scherrer U, Rexhaj E, Allemann Y, Sartori C, Rimoldi SF. Cardiovascular dysfunction in children conceived by assisted reproductive technologies. Eur Heart J. 2015;36(25):1583-1589. doi:10.1093/eurheartj/ehv145 25911649

[34] Fleming TP, Watkins AJ, Velazquez MA, . Origins of lifetime health around the time of conception: causes and consequences. Lancet. 2018;391(10132):1842-1852. doi:10.1016/S0140-6736(18)30312-X 29673874 PMC5975952

[35] Kindsfather AJ, Czekalski MA, Pressimone CA, Erisman MP, Mann MRW. Perturbations in imprinted methylation from assisted reproductive technologies but not advanced maternal age in mouse preimplantation embryos. Clin Epigenetics. 2019;11(1):162. doi:10.1186/s13148-019-0751-9 31767035 PMC6878706

[36] Choux C, Binquet C, Carmignac V, . The epigenetic control of transposable elements and imprinted genes in newborns is affected by the mode of conception: ART versus spontaneous conception without underlying infertility. Hum Reprod. 2018;33(2):331-340. doi:10.1093/humrep/dex366 29237055

[37] Li B, Chen S, Tang N, . Assisted reproduction causes reduced fetal growth associated with downregulation of paternally expressed imprinted genes that enhance fetal growth in mice. Biol Reprod. 2016;94(2):45. doi:10.1095/biolreprod.115.136051 26764349

[38] Chen W, Peng Y, Ma X, . Integrated multi-omics reveal epigenomic disturbance of assisted reproductive technologies in human offspring. EBioMedicine. 2020;61:103076. doi:10.1016/j.ebiom.2020.103076 33099088 PMC7585147

[39] Hattori H, Hiura H, Kitamura A, . Association of four imprinting disorders and ART. Clin Epigenetics. 2019;11(1):21. doi:10.1186/s13148-019-0623-3 30732658 PMC6367766

[40] Lazaraviciute G, Kauser M, Bhattacharya S, Haggarty P, Bhattacharya S. A systematic review and meta-analysis of DNA methylation levels and imprinting disorders in children conceived by IVF/ICSI compared with children conceived spontaneously. Hum Reprod Update. 2014;20(6):840-852. doi:10.1093/humupd/dmu033 24961233

[41] Huo Y, Yan ZQ, Yuan P, . Single-cell DNA methylation sequencing reveals epigenetic alterations in mouse oocytes superovulated with different dosages of gonadotropins. Clin Epigenetics. 2020;12(1):75. doi:10.1186/s13148-020-00866-w 32487258 PMC7268365

[42] Market-Velker BA, Fernandes AD, Mann MR. Side-by-side comparison of five commercial media systems in a mouse model: suboptimal in vitro culture interferes with imprint maintenance. Biol Reprod. 2010;83(6):938-950. doi:10.1095/biolreprod.110.085480 20702853

[43] Rodford JL, Torrens C, Siow RC, Mann GE, Hanson MA, Clough GF. Endothelial dysfunction and reduced antioxidant protection in an animal model of the developmental origins of cardiovascular disease. J Physiol. 2008;586(19):4709-4720. doi:10.1113/jphysiol.2008.156976 18669533 PMC2614035

[44] Giussani DA, Camm EJ, Niu Y, . Developmental programming of cardiovascular dysfunction by prenatal hypoxia and oxidative stress. PLoS One. 2012;7(2):e31017. doi:10.1371/journal.pone.0031017 22348036 PMC3278440

[45] Franco MdoC, Dantas APV, Akamine EH, . Enhanced oxidative stress as a potential mechanism underlying the programming of hypertension in utero. J Cardiovasc Pharmacol. 2002;40(4):501-509. doi:10.1097/00005344-200210000-00002 12352311

[46] Xiao D, Wang L, Huang X, Li Y, Dasgupta C, Zhang L. Protective effect of antenatal antioxidant on nicotine-induced heart ischemia-sensitive phenotype in rat offspring. PLoS One. 2016;11(2):e0150557. doi:10.1371/journal.pone.0150557 26918336 PMC4769226

[47] Yang H, Kuhn C, Kolben T, . Early life oxidative stress and long-lasting cardiovascular effects on offspring conceived by assisted reproductive technologies: a review. Int J Mol Sci. 2020;21(15):E5175. doi:10.3390/ijms21155175 32707756 PMC7432066

[48] Aljahdali A, Airina RKRI, Velazquez MA, . The duration of embryo culture after mouse IVF differentially affects cardiovascular and metabolic health in male offspring. Hum Reprod. 2020;35(11):2497-2514. doi:10.1093/humrep/deaa205 33020802 PMC7603862

[49] Pontesilli M, Painter RC, Grooten IJ, . Subfertility and assisted reproduction techniques are associated with poorer cardiometabolic profiles in childhood. Reprod Biomed Online. 2015;30(3):258-267. doi:10.1016/j.rbmo.2014.11.006 25592973

[50] Guo XY, Liu XM, Jin L, . Cardiovascular and metabolic profiles of offspring conceived by assisted reproductive technologies: a systematic review and meta-analysis. Fertil Steril. 2017;107(3):622-631.e5. doi:10.1016/j.fertnstert.2016.12.007 28104241

[51] Chen M, Wu L, Zhao J, . Altered glucose metabolism in mouse and humans conceived by IVF. Diabetes. 2014;63(10):3189-3198. doi:10.2337/db14-0103 24760136

[52] Gkourogianni A, Kosteria I, Telonis AG, . Plasma metabolomic profiling suggests early indications for predisposition to latent insulin resistance in children conceived by ICSI. PLoS One. 2014;9(4):e94001. doi:10.1371/journal.pone.0094001 24728198 PMC3984097

[53] Abdel-Maksoud MF, Eckel RH, Hamman RF, Hokanson JE. Risk of coronary heart disease is associated with triglycerides and high-density lipoprotein cholesterol in women and non-high-density lipoprotein cholesterol in men. J Clin Lipidol. 2012;6(4):374-381. doi:10.1016/j.jacl.2012.02.011 22836075

[54] Navar AM. The evolving story of triglycerides and coronary heart disease risk. JAMA. 2019;321(4):347-349. doi:10.1001/jama.2018.20044 30694301

[55] Ormazabal V, Nair S, Elfeky O, Aguayo C, Salomon C, Zuñiga FA. Association between insulin resistance and the development of cardiovascular disease. Cardiovasc Diabetol. 2018;17(1):122. doi:10.1186/s12933-018-0762-4 30170598 PMC6119242

