# Supplementary material for: Assessment of Cardiovascular Health of Children Ages 6 to 10 Years Conceived by Assisted Reproductive Technology
Source: JAMA Netw Open. 2021 Nov 4;4(11):e2132602. doi: 10.1001/jamanetworkopen.2021.32602 (PMC8569486; doi:10.1001/jamanetworkopen.2021.32602)
Supplement: Supplement. — eTable 1. Left Ventricular Structure and Function at Ages 6-10 Years by Assisted Reproductive Technology Fertilization Groups and Control Group eTable 2. Left Ventricular Structure and Function at Ages 6-10 Years by Embryo Groups and Control Group eFigure 1. Prevalence of Left Ventricular Hypertrophy (LVH), High Relative Wall Thickness (RWT), and Left Ventricular Remodeling by Fertilization Groups and Control Group eFigure 2. Prevalence of Left Ventricular Hypertrophy (LVH), High Relative Wall Thickness (RWT), and Left Ventricular Remodeling by Embryo Group and Control Group [file jamanetwopen-e2132602-s001.pdf]

Supplemental Online Content

Cui L, Zhao M, Zhang Z, et al. Assessment of cardiovascular health of children ages 6 to 10 years conceived by assisted reproductive technology. *JAMA Netw Open*. 2021;4(11):e2132602. doi:10.1001/jamanetworkopen.2021.32602

eTable 1. Left Ventricular Structure and Function at Ages 6-10 Years by Assisted Reproductive Technology Fertilization Groups and Control Group
eTable 2. Left Ventricular Structure and Function at Ages 6-10 Years by Embryo Groups and Control Group
eFigure 1. Prevalence of Left Ventricular Hypertrophy (LVH), High Relative Wall Thickness (RWT), and Left Ventricular Remodeling by Fertilization Groups and Control Group
eFigure 2. Prevalence of Left Ventricular Hypertrophy (LVH), High Relative Wall Thickness (RWT), and Left Ventricular Remodeling by Embryo Group and Control Group

This supplemental material has been provided by the authors to give readers additional information about their work.

© 2021 Cui L et al. *JAMA Network Open.*

eTable 1. Left Ventricular Structure and Function at Ages 6-10 Years by Assisted Reproductive Technology Fertilization Groups and Control Group

Characteristic	Controls (N=382)	ICSI (N=130)	IVF (N=243)	Adjusted *P* value*
**LV structure**				
LVDD, mm	3.81 (0.19) †§	3.88 (0.34)	3.85 (0.41)	0.087
LVSD, mm	24.26 (1.66) †§	25.32 (2.59)	25.20 (2.89)	<0.001
LVPWT, mm	5.75 (0.44) †§	6.31 (0.49)	6.26 (0.45)	<0.001
IVST, mm	5.60 (0.43) †§	6.38 (0.46)	6.32 (0.41)	<0.001
LVM, g	55.75 (10.59) †§	66.40 (14.03)	64.86 (15.01)	<0.001
LVMI, $g/m^{2.7}$	28.28 (3.54) †§	32.32 (4.91)	31.85 (5.03)	<0.001
RWT, mm	2.98 (0.14) †§	3.29 (0.34)	3.30 (0.42)	<0.001
**LV function**				
LVEF, %	66.70 (3.89) †§	64.68 (3.46)	64.53 (3.05)	<0.001
LVSF, %	36.37 (3.01) †§	34.86 (2.57)	34.63 (2.95)	<0.001
E/A ratio	2.21 (0.36) †§	1.66 (0.30)	1.65 (0.27)	<0.001

Data are presented as mean (SD)

ART, assisted reproductive technology; ICSI, intracytoplasmic sperm injection; IVF, in vitro fertilization; LV, left ventricular; LVDD, left ventricular end-diastolic internal dimension; LVSD, left ventricular end systolic internal dimension; LVPWT, left ventricular posterior wall thickness at end diastole; IVST, interventricular septal thickness; LVM, left ventricular mass; LVMI, left ventricular mass index; RWT, relative wall thickness; LVEF, left ventricular ejection fraction; LVSF, left ventricular shortening fraction; E/A, early (E) / late (A) mitral/tricuspid diastolic velocities

* Linear regression model was adjusted for gestational age at birth, birth weight, duration of breastfeeding, current weight status, BP status, and current lifestyle factors (fruit and vegetable intake, soft drink intake, physical activity, screen time and sleep duration)

† indicates *P*<0.05 for differences between ICSI group and control group

§ indicates *P*<0.05 for differences between IVF group and control group

© 2021 Cui L et al. *JAMA Network Open.*

eTable 2. Left Ventricular Structure and Function at Ages 6-10 Years by Embryo Groups and Control Group

Characteristic	Controls (N=382)	Fresh embryo (N=272)	Frozen embryo (N=107)	Adjusted *P* value*
**LV structure**				
LVDD, mm	3.81 (0.19) †	3.85 (0.41)	3.88 (0.34)	0.088
LVSD, mm	24.26 (1.66) †§	25.10 (2.87)	25.47 (2.62)	<0.001
LVPWT, mm	5.75 (0.44) †§	6.29 (0.50)	6.25 (0.38)	<0.001
IVST, mm	5.60 (0.43) †§	6.36 (0.46)	6.29 (0.36)	<0.001
LVM, g	55.75 (10.59) †§	65.37 (15.77)	65.17 (11.11)	<0.001
LVMI, $g/m^{2.7}$	28.28 (3.54) †§	31.83 (5.22)	32.29 (4.57)	<0.001
RWT, mm	2.98 (0.14) †§	3.32 (0.43)	3.26 (0.36)	<0.001
**LV function**				
LVEF, %	66.70 (3.89) †§	64.71 (3.31)	64.33 (2.85)	<0.001
LVSF, %	36.37 (3.01) †§	34.86 (2.47)	34.39 (3.52)	<0.001
E/A ratio	2.21 (0.36) †§	1.65 (0.28)	1.66 (0.28)	<0.001

Data are presented as mean (SD)

ART, assisted reproductive technology; ICSI, intracytoplasmic sperm injection; IVF, in vitro fertilization; LV, left ventricular; LVDD, left ventricular end-diastolic internal dimension; LVSD, left ventricular end systolic internal dimension; LVPWT, left ventricular posterior wall thickness at end diastole; IVST, interventricular septal thickness; LVM, left ventricular mass; LVMI, left ventricular mass index; RWT, relative wall thickness; LVEF, left ventricular ejection fraction; LVSF, left ventricular shortening fraction; E/A, early (E) / late (A) mitral/tricuspid diastolic velocities

* Linear regression model was adjusted for gestational age at birth, birth weight, duration of breastfeeding, current weight status, BP status, and current lifestyle factors (fruit and vegetable intake, soft drink intake, physical activity, screen time and sleep duration)

† indicates *P*<0.05 for differences between frozen embryo group and control group

§ indicates *P*<0.05 for differences between fresh embryo group and control group

© 2021 Cui L et al. *JAMA Network Open.*

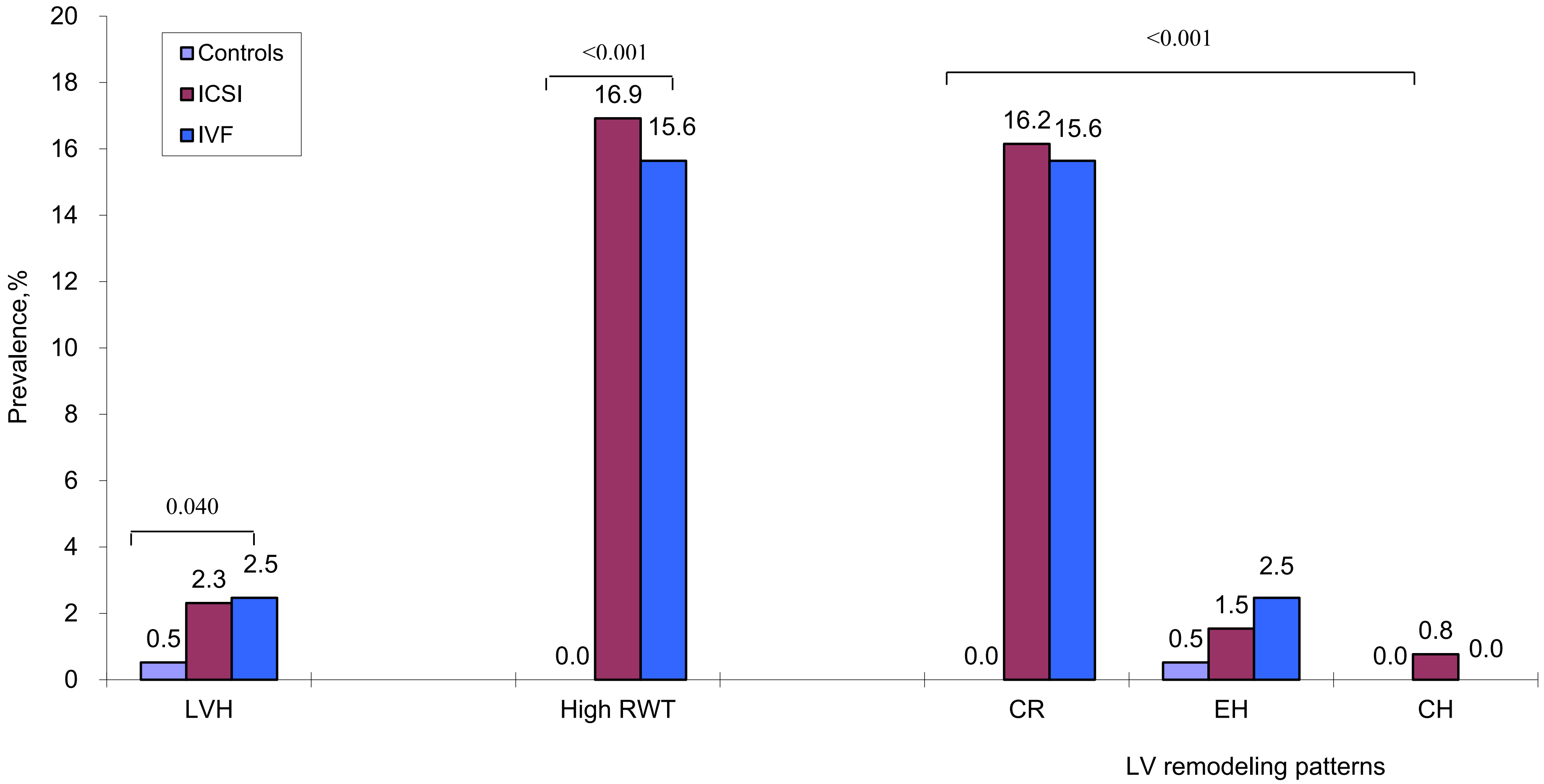

eFigure 1. Prevalence of Left Ventricular Hypertrophy (LVH), High Relative Wall Thickness (RWT), and Left Ventricular Remodeling by Fertilization Groups and Control Group
CR, concentric remodeling; EH, eccentric hypertrophy; CH, concentric hypertrophy; ICSI, intracytoplasmic sperm injection; IVF, in vitro fertilization

© 2021 Cui L et al. *JAMA Network Open.*

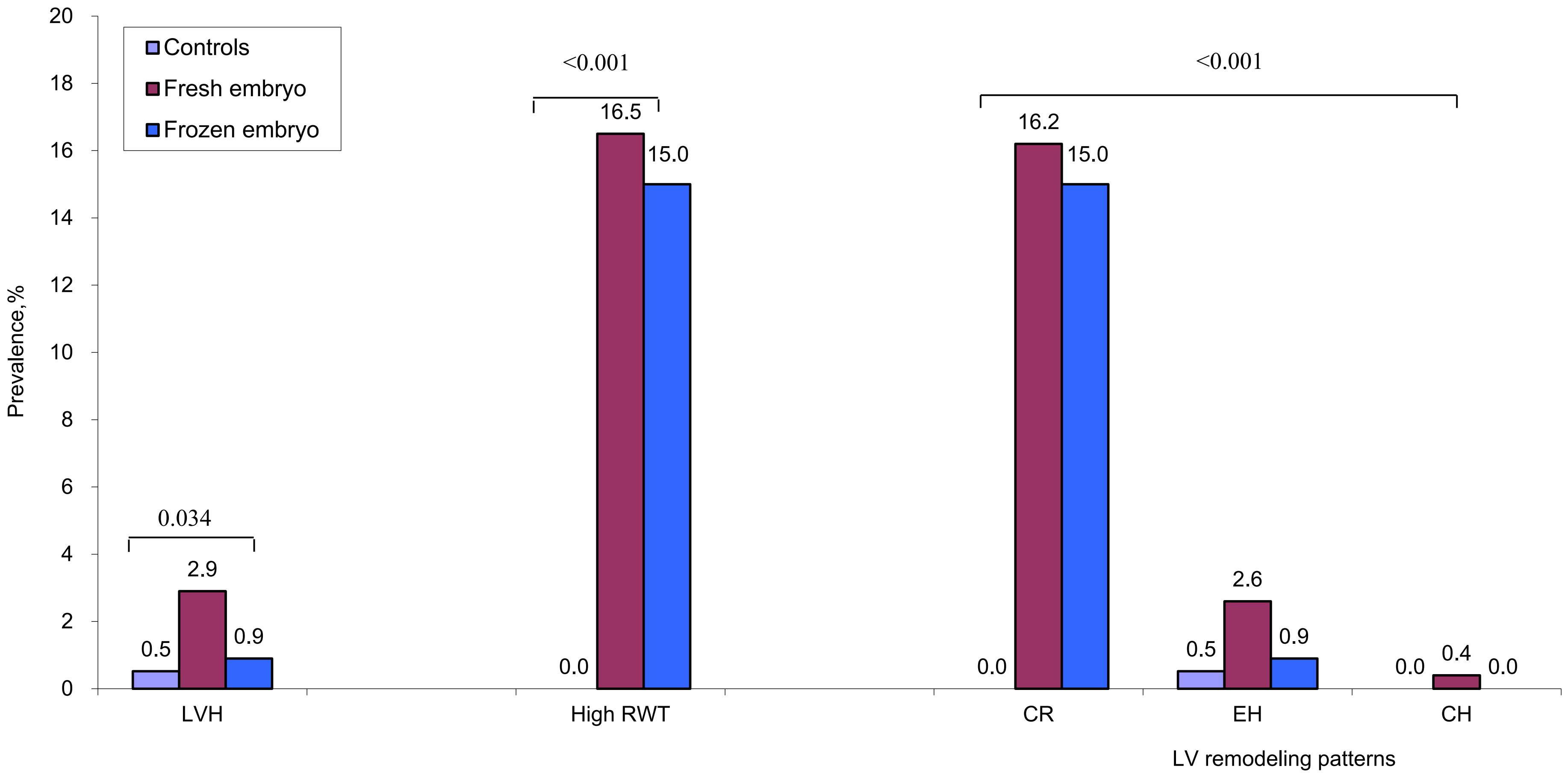

eFigure 2. Prevalence of Left Ventricular Hypertrophy (LVH), High Relative Wall Thickness (RWT), and Left Ventricular Remodeling by Embryo Group and Control Group CR, concentric remodeling; EH, eccentric hypertrophy; CH, concentric hypertrophy

© 2021 Cui L et al. *JAMA Network Open.*
